# 2-[2-(4-Nitro­phen­yl)hydrazinyl­idene]-1,3-diphenyl­propane-1,3-dione

**DOI:** 10.1107/S1600536811021143

**Published:** 2011-06-04

**Authors:** Carlos Bustos, Luis Alvarez-Thon, Daniela Barría, Maria Teresa Garland, Christian Sánchez

**Affiliations:** a Instituto de Ciencias Químicas, Universidad Austral de Chile, Avenida Los Robles s/n, Campus Isla Teja, Casilla 567, Valdivia, Chile; b Departamento de Ciencias Físicas, Universidad Andres Bello, Avenida República 220, Santiago de Chile, Chile; c Laboratorio de Cristalografía, Departamento de Física, Facultad de Ciencias Físicas y Matemáticas, Universidad de Chile, Santiago de Chile, Chile

## Abstract

In the mol­ecular structure of the title compound, C_21_H_15_N_3_O_4_, the inter­planar angle between the benzoyl units is 89.7 (1)°. The corresponding angles between the phenyl­hydrazono and the benzoyl groups are 31.4 (3) and 60.8 (2)°, respectively. In the crystal, a strong resonance-assisted intra­molecular hydrogen bond (N—H⋯O) and a weak intra­molecular hydrogen bond (C—H⋯N) strongly affect the observed conformation of the mol­ecule. The crystal packing is determined by a strong inter­molecular hydrogen bond (N—H⋯O), giving rise to a helical chain along the *a* axis. In addition, two weak inter­molecular contacts (C—H⋯O) are observed.

## Related literature

For details of the synthesis, see: Bustos *et al.* (2007 ▶, 2009 ▶); Yao (1964 ▶). For resonance-assisted hydrogen bonds and related structures, see: Bertolasi *et al.* (1993 ▶, 1994 ▶); Bustos *et al.* (2011 ▶).
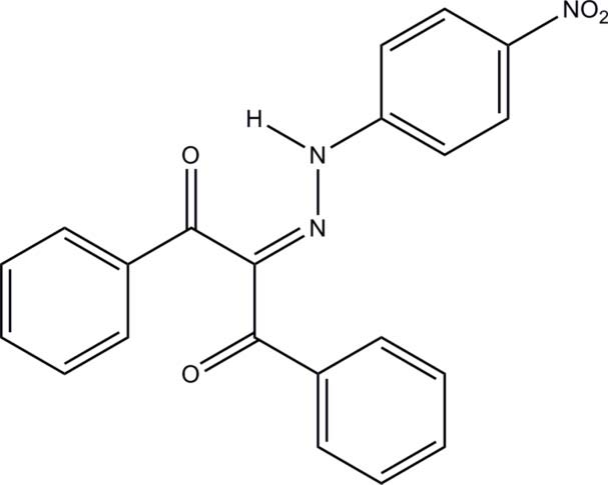


## Experimental

### 

#### Crystal data

C_21_H_15_N_3_O_4_
*M* *_r_* = 373.36
Orthorhombic, 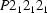

*a* = 8.2994 (7) Å
*b* = 8.6250 (7) Å
*c* = 25.018 (2) Å
*V* = 1790.9 (3) Å^3^
*Z* = 4
Mo *K*α radiation
μ = 0.10 mm^−1^
*T* = 150 K
0.30 × 0.28 × 0.12 mm

#### Data collection

Bruker D8 Discover with SMART CCD area-detector diffractometer
13939 measured reflections
2114 independent reflections
1826 reflections with *I* > 2σ(*I*)
*R*_int_ = 0.034

#### Refinement

*R*[*F* ^2^ > 2σ(*F* ^2^)] = 0.034
*wR*(*F* ^2^) = 0.084
*S* = 1.00
2114 reflections
257 parameters
H atoms treated by a mixture of independent and constrained refinement
Δρ_max_ = 0.19 e Å^−3^
Δρ_min_ = −0.16 e Å^−3^

### 

Data collection: *SMART* (Bruker, 2001 ▶); cell refinement: *SAINT* (Bruker, 2000 ▶); data reduction: *SAINT*; program(s) used to solve structure: *SHELXS97* (Sheldrick, 2008 ▶); program(s) used to refine structure: *SHELXL97* (Sheldrick, 2008 ▶); molecular graphics: *XP* in *SHELXTL/PC* (Sheldrick, 2008 ▶); software used to prepare material for publication: *PLATON* (Spek, 2009 ▶) and *Mercury* (Macrae *et al.*, 2006 ▶).

## Supplementary Material

Crystal structure: contains datablock(s) global, I. DOI: 10.1107/S1600536811021143/im2291sup1.cif

Structure factors: contains datablock(s) I. DOI: 10.1107/S1600536811021143/im2291Isup2.hkl

Supplementary material file. DOI: 10.1107/S1600536811021143/im2291Isup3.cml

Additional supplementary materials:  crystallographic information; 3D view; checkCIF report

## Figures and Tables

**Table 1:** Hydrogen-bond geometry (Å, °)

*D*—H⋯*A*	*D*—H	H⋯*A*	*D*⋯*A*	*D*—H⋯*A*
N2—H1⋯O2	0.96 (3)	2.25 (3)	2.793 (2)	115.3 (19)
N2—H1⋯O1^i^	0.96 (3)	2.15 (2)	2.956 (2)	142 (2)
C5—H5⋯O3^ii^	0.95	2.60	3.419 (3)	145
C15—H15⋯N1	0.95	2.42	2.849 (3)	107
C20—H20⋯O2^iii^	0.95	2.44	3.352 (2)	161

Symmetry codes: (i) 
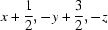
; (ii) 
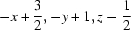
; (iii) 
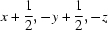
.

**Fig. 1.**
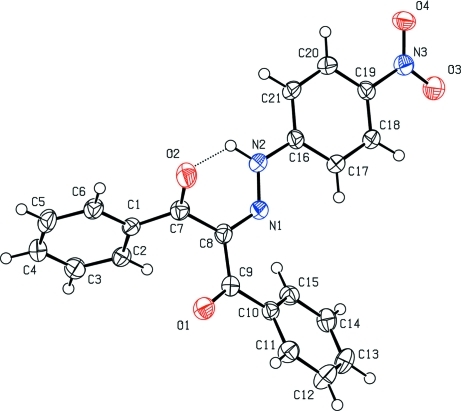
View of the title compound with the atom numbering scheme. Displacement ellipsoids for non-H atoms are drawn at the 50% probability level. The strong intramolecular hydrogen bond (N2–H1···O2) is depicted with dashed lines.

**Fig. 2.**
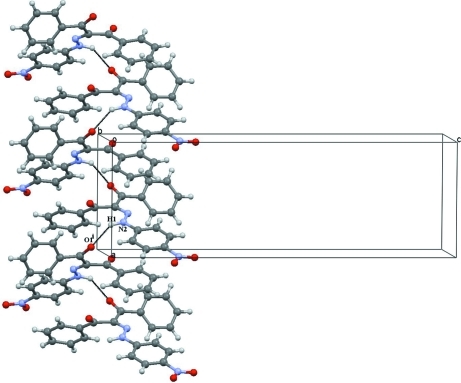
Perspective view along the b axis showing the formation of a zigzag chain, along the a axis, linked by strong intermolecular hydrogen bonds, N2–H1···O1i (dashed lines), [symmetry code: (i) x + 1/2, -y + 3/2, -z].

**Fig. 3.**
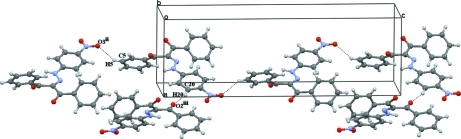
Perspective view along the b axis showing the formation of a chain, along the c axis, linked by weak intermolecular contacts (C5–H5···O3ii and C20–H20···O2iii), [symmetry codes: (ii) -x + 3/2, -y + 1, -1/2 + z; (iii) x + 1/2, -y + 1/2, -z].

## supplementary crystallographic information

### Comment

The crystal structure of 2-[(4-nitro-phenyl)-hydrazono]-1,3-diphenyl-propane-1,3-dione is reported. This compound belongs to a family that contain a six-membered π–conjugated ring closed *via* strong intramolecular resonance assisted hydrogen bonds, N–H···O,  RAHB (Resonance Assisted Hydrogen Bond) which, *inter alia*, could have remarkable importance as bistate in molecular switches (Bertolasi *et al.*, 1993; Bertolasi *et al.*, 1994; Bustos *et al.*, 2011). On the other hand, it is well known that the phenyl diazonium salts are capable of coupling with a series of β-diketonate anions to give β-diketohydrazones containing N–H···O moieties (Yao, 1964; Bustos *et al.*, 2007; Bustos *et al.*, 2009). Using this reaction (Yao, 1964) we have prepared the title compound.

The molecular structure of the title compound, C_21_H_15_N_3_O_4_, exhibits a strong intramolecular hydrogen bond (N2–H1···O2) and a weak intramolecular hydrogen bond (C15–H15···N1) (Fig. 1 and Tab. 1). In the crystal structure, strong intermolecular hydrogen bonds (N2–H1···O1^i^) link the molecules into helical chains along the *a* axis, which may be the reason why the title compound crystallizes in the chiral space group *P*2_1_2_1_2_1_ (see Fig. 2 and Tab. 1), [symmetry code: (i) *x* + 1/2, -*y* + 3/2, -*z*]. On the other hand, weak intermolecular contacts of the type C5–H5···O3^ii^ and C20–H20···O2^iii^, further stabilize the crystal packing to construct the entire three-dimensional network, see Fig. 3 and Tab. 1, [symmetry codes: (ii) -*x* + 3/2, -*y* + 1, -1/2 + *z*; (iii) *x* + 1/2, -*y* + 1/2, -*z*]. The interplanar angle between the benzoyl moieties is 89.7 (1)°. The corresponding angles between the phenyl-hydrazono and the benzoyl groups, are 31.4 (3)° and 60.8 (2)°, respectively.

### Experimental

In a 500 ml flask, 2.24 g (0.01 mole) of 1,3-diphenylpropane-1,3-dione were dissolved in 100 ml of a ethanolic solution that contained 0.4 g (0.01 mole) of sodium hydroxide and 3.65 g (0.045 mole) of sodium acetate. The resulting β-diketonate solution was diluted with water to a final volume of about 220 ml, stirred and cooled at 268 K. In another 50 ml beaker a diazonium ion solution was prepared adding 1.39 g (0.01 mole) of 4-nitroaniline (99%) in 8 ml of hydrochloric acid (5 mol/*L*), cooling at 268 K, and adding a saturated aqueous solution containing 0.69 g (0.01 mole) of sodium nitrite. The diazonium salt solution was then added dropwise with vigorous stirring at 268 K into the β-diketonate solution. During the addition a yellow solid precipitate of the title compound was formed which was filtered by suction, washed with an abundant quantity of water and dried in the vacuum at 313 K (Yield: 91% of crude product). Single crystals suitable for X-ray studies were obtained by recrystallization from ethanol.

### Refinement

All hydrogen atoms were found in difference Fourier maps. The hydrogen attached to N2 was refined freely against the diffraction data, but all other H atoms were placed in geometrically idealized positions and constrained to ride on their parent atoms, with C—H = 0.95 Å and *U*_iso_(H) = 1.2*U*_eq_(C). In the absence of significant anomalous dispersion effects Friedel pairs were also merged.

### Crystal data

**Table:**

C_21_H_15_N_3_O_4_	*F*(000) = 776
*M**_r_* = 373.36	*D*_x_ = 1.385 Mg m^−^^3^
Orthorhombic, *P*2_1_2_1_2_1_	Mo *K*α radiation, λ = 0.71073 Å
Hall symbol: P 2ac 2ab	Cell parameters from 999 reflections
*a* = 8.2994 (7) Å	θ = 1.6–26.4°
*b* = 8.6250 (7) Å	µ = 0.10 mm^−^^1^
*c* = 25.018 (2) Å	*T* = 150 K
*V* = 1790.9 (3) Å^3^	Polyhedron, yellow
*Z* = 4	0.30 × 0.28 × 0.12 mm

### Data collection

**Table:**

Bruker D8 Discover with SMART CCD area-detector diffractometer	1826 reflections with *I* > 2σ(*I*)
Radiation source: fine-focus sealed tube	*R*_int_ = 0.034
graphite	θ_max_ = 26.4°, θ_min_ = 1.6°
φ and ω scans	*h* = −10→10
13939 measured reflections	*k* = −10→10
2114 independent reflections	*l* = −31→31

### Refinement

**Table:**

Refinement on *F*^2^	Primary atom site location: structure-invariant direct methods
Least-squares matrix: full	Secondary atom site location: difference Fourier map
*R*[*F*^2^ > 2σ(*F*^2^)] = 0.034	Hydrogen site location: inferred from neighbouring sites
*wR*(*F*^2^) = 0.084	H atoms treated by a mixture of independent and constrained refinement
*S* = 1.00	*w* = 1/[σ^2^(*F*_o_^2^) + (0.0526*P*)^2^] where *P* = (*F*_o_^2^ + 2*F*_c_^2^)/3
2114 reflections	(Δ/σ)_max_ < 0.001
257 parameters	Δρ_max_ = 0.19 e Å^−^^3^
0 restraints	Δρ_min_ = −0.16 e Å^−^^3^

### Special details

**Table:**

Geometry. Bond distances, angles *etc*. have been calculated using the rounded fractional coordinates. All su's are estimated from the variances of the (full) variance-covariance matrix. The cell e.s.d.'s are taken into account in the estimation of distances, angles and torsion angles
Refinement. Refinement on *F*^2^ for ALL reflections except those flagged by the user for potential systematic errors. Weighted *R*-factors *wR* and all goodnesses of fit *S* are based on *F*^2^, conventional *R*-factors *R* are based on *F*, with *F* set to zero for negative *F*^2^. The observed criterion of *F*^2^ > σ(*F*^2^) is used only for calculating -*R*-factor-obs *etc*. and is not relevant to the choice of reflections for refinement. *R*-factors based on *F*^2^ are statistically about twice as large as those based on *F*, and *R*-factors based on ALL data will be even larger.

### Fractional atomic coordinates and isotropic or equivalent isotropic displacement parameters (Å^2^)

**Table:**

	*x*	*y*	*z*	*U*_iso_*/*U*_eq_	
O1	0.44608 (18)	0.97963 (18)	0.03744 (5)	0.0389 (5)	
O2	0.5740 (2)	0.61910 (18)	−0.03732 (6)	0.0466 (6)	
O3	1.0083 (2)	0.13731 (18)	0.24696 (6)	0.0465 (5)	
O4	1.04378 (19)	−0.02554 (17)	0.18195 (6)	0.0388 (5)	
N1	0.6619 (2)	0.65447 (19)	0.07404 (7)	0.0296 (5)	
N2	0.7292 (2)	0.5252 (2)	0.05629 (7)	0.0334 (6)	
N3	0.9963 (2)	0.1000 (2)	0.19940 (7)	0.0337 (6)	
C1	0.6762 (2)	0.8692 (2)	−0.05394 (8)	0.0276 (6)	
C2	0.7655 (2)	0.9941 (3)	−0.03441 (8)	0.0316 (6)	
C3	0.8288 (3)	1.1021 (3)	−0.06939 (9)	0.0386 (7)	
C4	0.8022 (3)	1.0870 (3)	−0.12373 (9)	0.0398 (8)	
C5	0.7127 (3)	0.9651 (3)	−0.14324 (8)	0.0399 (7)	
C6	0.6502 (3)	0.8551 (3)	−0.10873 (8)	0.0339 (7)	
C7	0.6156 (3)	0.7439 (2)	−0.01897 (8)	0.0302 (6)	
C8	0.6125 (2)	0.7616 (2)	0.04127 (7)	0.0289 (6)	
C9	0.5292 (2)	0.8954 (2)	0.06604 (7)	0.0282 (6)	
C10	0.5405 (2)	0.9243 (2)	0.12510 (7)	0.0272 (6)	
C11	0.4112 (3)	0.9999 (3)	0.14872 (8)	0.0353 (7)	
C12	0.4119 (3)	1.0314 (3)	0.20280 (8)	0.0436 (8)	
C13	0.5415 (3)	0.9878 (3)	0.23385 (8)	0.0404 (7)	
C14	0.6709 (3)	0.9125 (3)	0.21065 (8)	0.0348 (7)	
C15	0.6721 (3)	0.8826 (2)	0.15632 (8)	0.0303 (6)	
C16	0.7912 (3)	0.4192 (2)	0.09311 (8)	0.0300 (6)	
C17	0.7726 (3)	0.4409 (2)	0.14817 (8)	0.0331 (7)	
C18	0.8404 (3)	0.3353 (2)	0.18280 (8)	0.0331 (6)	
C19	0.9223 (2)	0.2091 (2)	0.16230 (8)	0.0289 (6)	
C20	0.9391 (3)	0.1843 (2)	0.10811 (8)	0.0336 (7)	
C21	0.8739 (3)	0.2911 (2)	0.07362 (8)	0.0340 (7)	
H1	0.752 (3)	0.511 (3)	0.0191 (10)	0.052 (7)*	
H2	0.78270	1.00480	0.00290	0.0380*	
H3	0.89040	1.18660	−0.05610	0.0460*	
H4	0.84600	1.16120	−0.14770	0.0480*	
H5	0.69380	0.95640	−0.18060	0.0480*	
H6	0.58980	0.77020	−0.12230	0.0410*	
H11	0.32160	1.03010	0.12750	0.0420*	
H12	0.32290	1.08320	0.21870	0.0520*	
H13	0.54190	1.00940	0.27110	0.0480*	
H14	0.75950	0.88110	0.23210	0.0420*	
H15	0.76270	0.83370	0.14040	0.0360*	
H17	0.71400	0.52720	0.16150	0.0400*	
H18	0.83090	0.34900	0.22030	0.0400*	
H20	0.99430	0.09560	0.09500	0.0400*	
H21	0.88550	0.27720	0.03610	0.0410*	

### Atomic displacement parameters (Å^2^)

**Table:**

	*U*^11^	*U*^22^	*U*^33^	*U*^12^	*U*^13^	*U*^23^
O1	0.0424 (8)	0.0457 (9)	0.0285 (8)	0.0103 (8)	−0.0056 (7)	0.0023 (7)
O2	0.0727 (12)	0.0396 (9)	0.0274 (8)	−0.0174 (9)	−0.0043 (8)	−0.0027 (7)
O3	0.0627 (11)	0.0466 (9)	0.0301 (8)	0.0086 (9)	−0.0044 (7)	0.0013 (8)
O4	0.0404 (8)	0.0306 (8)	0.0454 (9)	0.0061 (7)	0.0028 (7)	−0.0011 (7)
N1	0.0347 (10)	0.0272 (9)	0.0269 (9)	−0.0012 (8)	0.0029 (8)	−0.0008 (8)
N2	0.0467 (11)	0.0303 (10)	0.0233 (9)	0.0025 (9)	0.0042 (8)	−0.0018 (8)
N3	0.0341 (10)	0.0334 (10)	0.0337 (10)	−0.0014 (8)	0.0013 (8)	0.0006 (8)
C1	0.0298 (11)	0.0284 (11)	0.0245 (10)	0.0024 (9)	0.0007 (8)	−0.0015 (9)
C2	0.0380 (11)	0.0327 (11)	0.0241 (10)	0.0008 (10)	−0.0010 (9)	−0.0020 (10)
C3	0.0443 (13)	0.0333 (12)	0.0382 (12)	−0.0054 (11)	0.0038 (11)	−0.0016 (10)
C4	0.0497 (14)	0.0363 (13)	0.0333 (12)	−0.0016 (11)	0.0087 (11)	0.0063 (10)
C5	0.0500 (14)	0.0477 (14)	0.0221 (10)	−0.0011 (12)	0.0013 (10)	0.0009 (10)
C6	0.0390 (12)	0.0362 (12)	0.0264 (10)	−0.0011 (11)	−0.0004 (9)	−0.0039 (9)
C7	0.0324 (11)	0.0323 (11)	0.0259 (11)	−0.0023 (10)	−0.0030 (9)	−0.0023 (10)
C8	0.0325 (11)	0.0302 (11)	0.0240 (10)	−0.0036 (9)	0.0001 (9)	0.0007 (9)
C9	0.0273 (10)	0.0313 (11)	0.0259 (10)	−0.0028 (9)	−0.0010 (8)	0.0017 (9)
C10	0.0298 (10)	0.0256 (10)	0.0262 (10)	−0.0028 (9)	0.0012 (9)	0.0013 (9)
C11	0.0324 (11)	0.0431 (13)	0.0304 (11)	0.0050 (11)	−0.0039 (9)	−0.0004 (10)
C12	0.0352 (12)	0.0642 (16)	0.0315 (11)	0.0047 (12)	0.0038 (10)	−0.0072 (12)
C13	0.0428 (13)	0.0565 (14)	0.0218 (10)	−0.0043 (12)	0.0000 (9)	−0.0029 (10)
C14	0.0349 (12)	0.0398 (12)	0.0296 (11)	−0.0040 (10)	−0.0066 (9)	0.0035 (10)
C15	0.0322 (11)	0.0285 (11)	0.0302 (11)	−0.0016 (9)	−0.0013 (9)	−0.0015 (9)
C16	0.0377 (12)	0.0256 (11)	0.0268 (10)	−0.0048 (9)	0.0039 (9)	0.0014 (9)
C17	0.0433 (13)	0.0286 (11)	0.0275 (10)	0.0024 (10)	0.0073 (9)	−0.0012 (9)
C18	0.0451 (12)	0.0300 (11)	0.0241 (10)	−0.0003 (10)	0.0056 (10)	−0.0012 (9)
C19	0.0337 (11)	0.0242 (10)	0.0288 (10)	−0.0039 (9)	0.0021 (9)	0.0014 (9)
C20	0.0420 (12)	0.0271 (11)	0.0318 (11)	−0.0001 (10)	0.0055 (10)	−0.0035 (9)
C21	0.0470 (13)	0.0309 (11)	0.0242 (10)	−0.0020 (11)	0.0059 (10)	−0.0056 (9)

### Geometric parameters (Å, °)

**Table:**

O1—C9	1.231 (2)	C13—C14	1.383 (3)
O2—C7	1.220 (2)	C14—C15	1.384 (3)
O3—N3	1.237 (2)	C16—C21	1.389 (3)
O4—N3	1.232 (2)	C16—C17	1.399 (3)
N1—N2	1.324 (2)	C17—C18	1.377 (3)
N1—C8	1.302 (2)	C18—C19	1.382 (3)
N2—C16	1.396 (3)	C19—C20	1.380 (3)
N3—C19	1.457 (3)	C20—C21	1.373 (3)
N2—H1	0.96 (3)	C2—H2	0.9500
C1—C6	1.393 (3)	C3—H3	0.9500
C1—C7	1.479 (3)	C4—H4	0.9500
C1—C2	1.396 (3)	C5—H5	0.9500
C2—C3	1.382 (3)	C6—H6	0.9500
C3—C4	1.383 (3)	C11—H11	0.9500
C4—C5	1.377 (4)	C12—H12	0.9500
C5—C6	1.384 (3)	C13—H13	0.9500
C7—C8	1.515 (3)	C14—H14	0.9500
C8—C9	1.481 (2)	C15—H15	0.9500
C9—C10	1.501 (2)	C17—H17	0.9500
C10—C11	1.388 (3)	C18—H18	0.9500
C10—C15	1.390 (3)	C20—H20	0.9500
C11—C12	1.380 (3)	C21—H21	0.9500
C12—C13	1.379 (3)		
			
O1···C1	3.128 (2)	C15···C19^xi^	3.502 (3)
O1···C2	3.205 (2)	C15···O4^xi^	3.249 (3)
O1···C7^i^	3.384 (3)	C15···N1	2.849 (3)
O1···O2^i^	3.203 (2)	C16···C6^iii^	3.580 (3)
O1···N2^i^	2.956 (2)	C18···O3^xii^	3.384 (2)
O2···N2	2.793 (2)	C19···C15^vi^	3.502 (3)
O2···C2^i^	3.276 (2)	C19···C14^vi^	3.516 (3)
O2···C20^ii^	3.352 (2)	C20···O2^viii^	3.352 (2)
O2···N1	2.896 (2)	C3···H21^xi^	3.0800
O2···O1^iii^	3.203 (2)	C4···H13^xiii^	3.0500
O3···C18^iv^	3.384 (2)	C5···H13^xiii^	2.9600
O3···C5^v^	3.419 (3)	C7···H1	2.50 (3)
O4···C15^vi^	3.249 (3)	C8···H15	2.8400
O4···C11^vii^	3.168 (3)	C8···H2	2.7000
O4···C12^vii^	3.138 (3)	C9···H2	2.7900
O4···C14^vi^	3.222 (3)	C11···H4^xiv^	2.9700
O1···H1^i^	2.15 (2)	C12···H4^xiv^	3.0400
O1···H21^i^	2.8300	C15···H17	3.0900
O1···H11	2.5200	H1···O2	2.25 (3)
O2···H2^i^	2.7800	H1···C7	2.50 (3)
O2···H20^ii^	2.4400	H1···H21	2.3400
O2···H1	2.25 (3)	H1···O1^iii^	2.15 (2)
O2···H6	2.5000	H2···C8	2.7000
O3···H14^iv^	2.9000	H2···C9	2.7900
O3···H12^vii^	2.7400	H2···O2^iii^	2.7800
O3···H18	2.4400	H3···H21^xi^	2.4400
O3···H5^v^	2.6000	H4···C11^xv^	2.9700
O4···H11^vii^	2.7200	H4···C12^xv^	3.0400
O4···H12^vii^	2.6600	H5···O3^ix^	2.6000
O4···H14^vi^	2.7900	H5···H13^xiii^	2.5200
O4···H15^vi^	2.8300	H6···O2	2.5000
O4···H20	2.4500	H6···O4^ii^	2.6100
O4···H6^viii^	2.6100	H11···O1	2.5200
O4···H18^iv^	2.8700	H11···O4^x^	2.7200
N1···O2	2.896 (2)	H12···O3^x^	2.7400
N1···C15	2.849 (3)	H12···O4^x^	2.6600
N2···O1^iii^	2.956 (2)	H12···N3^x^	2.7600
N2···O2	2.793 (2)	H13···C4^xvi^	3.0500
N3···C14^vi^	3.160 (3)	H13···C5^xvi^	2.9600
N1···H15	2.4200	H13···H5^xvi^	2.5200
N1···H17	2.4900	H14···O4^xi^	2.7900
N3···H12^vii^	2.7600	H14···N3^xi^	2.8500
N3···H14^vi^	2.8500	H14···O3^xii^	2.9000
C1···O1	3.128 (2)	H15···O4^xi^	2.8300
C2···O1	3.205 (2)	H15···N1	2.4200
C2···C9	3.299 (3)	H15···C8	2.8400
C2···O2^iii^	3.276 (2)	H17···N1	2.4900
C5···O3^ix^	3.419 (3)	H17···C15	3.0900
C6···C16^i^	3.580 (3)	H18···O3	2.4400
C7···O1^iii^	3.384 (3)	H18···O4^xii^	2.8700
C9···C2	3.299 (3)	H20···O4	2.4500
C11···O4^x^	3.168 (3)	H20···O2^viii^	2.4400
C12···O4^x^	3.138 (3)	H21···C3^vi^	3.0800
C14···O4^xi^	3.222 (3)	H21···H1	2.3400
C14···N3^xi^	3.160 (3)	H21···H3^vi^	2.4400
C14···C19^xi^	3.516 (3)	H21···O1^iii^	2.8300
			
N2—N1—C8	121.30 (17)	C16—C17—C18	119.07 (18)
N1—N2—C16	119.06 (17)	C17—C18—C19	119.23 (18)
O3—N3—O4	122.94 (17)	N3—C19—C20	118.89 (16)
O3—N3—C19	118.60 (16)	N3—C19—C18	118.65 (18)
O4—N3—C19	118.46 (16)	C18—C19—C20	122.45 (18)
N1—N2—H1	121.1 (16)	C19—C20—C21	118.27 (18)
C16—N2—H1	119.0 (15)	C16—C21—C20	120.51 (19)
C2—C1—C6	119.61 (19)	C1—C2—H2	120.00
C2—C1—C7	122.52 (18)	C3—C2—H2	120.00
C6—C1—C7	117.76 (18)	C2—C3—H3	120.00
C1—C2—C3	120.03 (19)	C4—C3—H3	120.00
C2—C3—C4	119.9 (2)	C3—C4—H4	120.00
C3—C4—C5	120.4 (2)	C5—C4—H4	120.00
C4—C5—C6	120.3 (2)	C4—C5—H5	120.00
C1—C6—C5	119.7 (2)	C6—C5—H5	120.00
O2—C7—C8	117.28 (17)	C1—C6—H6	120.00
O2—C7—C1	121.23 (18)	C5—C6—H6	120.00
C1—C7—C8	121.38 (16)	C10—C11—H11	120.00
N1—C8—C9	115.89 (16)	C12—C11—H11	120.00
N1—C8—C7	123.35 (16)	C11—C12—H12	120.00
C7—C8—C9	120.18 (15)	C13—C12—H12	120.00
O1—C9—C10	120.60 (16)	C12—C13—H13	120.00
C8—C9—C10	120.79 (15)	C14—C13—H13	120.00
O1—C9—C8	118.57 (16)	C13—C14—H14	120.00
C11—C10—C15	119.33 (17)	C15—C14—H14	120.00
C9—C10—C11	116.66 (16)	C10—C15—H15	120.00
C9—C10—C15	123.99 (16)	C14—C15—H15	120.00
C10—C11—C12	120.5 (2)	C16—C17—H17	120.00
C11—C12—C13	120.1 (2)	C18—C17—H17	120.00
C12—C13—C14	119.83 (19)	C17—C18—H18	120.00
C13—C14—C15	120.4 (2)	C19—C18—H18	120.00
C10—C15—C14	119.9 (2)	C19—C20—H20	121.00
C17—C16—C21	120.44 (18)	C21—C20—H20	121.00
N2—C16—C17	121.43 (18)	C16—C21—H21	120.00
N2—C16—C21	118.12 (18)	C20—C21—H21	120.00
			
C8—N1—N2—C16	175.19 (18)	N1—C8—C9—C10	15.3 (2)
N2—N1—C8—C7	4.7 (3)	C7—C8—C9—O1	9.2 (3)
N2—N1—C8—C9	175.89 (16)	C7—C8—C9—C10	−173.16 (16)
N1—N2—C16—C17	5.0 (3)	O1—C9—C10—C11	26.0 (3)
N1—N2—C16—C21	−174.39 (19)	O1—C9—C10—C15	−152.83 (18)
O3—N3—C19—C18	−12.6 (3)	C8—C9—C10—C11	−151.62 (18)
O3—N3—C19—C20	166.19 (18)	C8—C9—C10—C15	29.6 (3)
O4—N3—C19—C18	167.23 (18)	C9—C10—C11—C12	−179.9 (2)
O4—N3—C19—C20	−14.0 (3)	C15—C10—C11—C12	−1.0 (3)
C6—C1—C2—C3	−0.6 (3)	C9—C10—C15—C14	−179.29 (19)
C7—C1—C2—C3	175.4 (2)	C11—C10—C15—C14	1.9 (3)
C2—C1—C6—C5	−0.2 (3)	C10—C11—C12—C13	0.0 (4)
C7—C1—C6—C5	−176.4 (2)	C11—C12—C13—C14	0.1 (4)
C2—C1—C7—O2	−161.6 (2)	C12—C13—C14—C15	0.9 (4)
C2—C1—C7—C8	14.5 (3)	C13—C14—C15—C10	−1.9 (3)
C6—C1—C7—O2	14.5 (3)	N2—C16—C17—C18	−178.0 (2)
C6—C1—C7—C8	−169.39 (19)	C21—C16—C17—C18	1.4 (3)
C1—C2—C3—C4	0.6 (3)	N2—C16—C21—C20	179.2 (2)
C2—C3—C4—C5	0.1 (4)	C17—C16—C21—C20	−0.3 (4)
C3—C4—C5—C6	−0.9 (4)	C16—C17—C18—C19	−1.3 (3)
C4—C5—C6—C1	0.9 (4)	C17—C18—C19—N3	178.75 (19)
O2—C7—C8—N1	42.0 (3)	C17—C18—C19—C20	0.0 (3)
O2—C7—C8—C9	−128.9 (2)	N3—C19—C20—C21	−177.62 (19)
C1—C7—C8—N1	−134.3 (2)	C18—C19—C20—C21	1.1 (3)
C1—C7—C8—C9	54.9 (3)	C19—C20—C21—C16	−1.0 (3)
N1—C8—C9—O1	−162.31 (17)		

Symmetry codes: (i) *x*−1/2, −*y*+3/2, −*z*; (ii) *x*−1/2, −*y*+1/2, −*z*; (iii) *x*+1/2, −*y*+3/2, −*z*; (iv) −*x*+2, *y*−1/2, −*z*+1/2; (v) −*x*+3/2, −*y*+1, *z*+1/2; (vi) *x*, *y*−1, *z*; (vii) *x*+1, *y*−1, *z*; (viii) *x*+1/2, −*y*+1/2, −*z*; (ix) −*x*+3/2, −*y*+1, *z*−1/2; (x) *x*−1, *y*+1, *z*; (xi) *x*, *y*+1, *z*; (xii) −*x*+2, *y*+1/2, −*z*+1/2; (xiii) −*x*+3/2, −*y*+2, *z*−1/2; (xiv) *x*−1/2, −*y*+5/2, −*z*; (xv) *x*+1/2, −*y*+5/2, −*z*; (xvi) −*x*+3/2, −*y*+2, *z*+1/2.

### Hydrogen-bond geometry (Å, °)

**Table:**

*D*—H···*A*	*D*—H	H···*A*	*D*···*A*	*D*—H···*A*
N2—H1···O2	0.96 (3)	2.25 (3)	2.793 (2)	115.3 (19)
N2—H1···O1^iii^	0.96 (3)	2.15 (2)	2.956 (2)	142 (2)
C5—H5···O3^ix^	0.95	2.60	3.419 (3)	145
C15—H15···N1	0.95	2.42	2.849 (3)	107
C20—H20···O2^viii^	0.95	2.44	3.352 (2)	161

Symmetry codes: (iii) *x*+1/2, −*y*+3/2, −*z*; (ix) −*x*+3/2, −*y*+1, *z*−1/2; (viii) *x*+1/2, −*y*+1/2, −*z*.
